# Nonlinear Analysis of the Hand and Foot Force-Time Profiles in the Four Competitive Swimming Strokes

**DOI:** 10.5114/jhk/172616

**Published:** 2023-11-28

**Authors:** Raul Filipe Bartolomeu, Pedro Rodrigues, Kamil Sokołowski, Marek Strzała, Catarina Costa Santos, Mário Jorge Costa, Tiago Manuel Barbosa

**Affiliations:** 1 Department of Sports Sciences, Polytechnic of Guarda, Guarda, Portugal.; 2 Department of Sport Sciences and Physical Education, Instituto Politécnico de Bragança, Bragança, Portugal.; 3 Research Center in Sports Sciences, Health and Human Development (CIDESD), Vila Real, Portugal.; 4 Department of Sport Sciences, University of Beira Interior, Covilhã, Portugal.; 5 Department of Water Sports, Faculty of Physical Education and Sport, University of Physical Education, Kraków, Poland.; 6 Faculty of Sport, University of Porto, Porto, Portugal.; 7 Centre of Research, Education, Innovation and Intervention in Sport (CIFI2D), Faculty of Sport, University of Porto, Porto, Portugal.; 8 Porto Biomechanics Laboratory (LABIOMEP-UP), University of Porto, Porto, Portugal.

**Keywords:** hand and foot force, segmental actions, fractal dimension, entropy, nonlinear analysis

## Abstract

Human locomotion on water depends on the force produced by the swimmer to propel the body forward. Performance of highly complex motor tasks like swimming can yield minor variations that only nonlinear analysis can be sensitive enough to detect. The purpose of the present study was to examine the nonlinear properties of the hand/feet forces and describe their variations across the four competitive swimming strokes performing segmental and full-body swimming. Swimmers performed all-out bouts of 25 m in the four swimming strokes, swimming the full-body stroke, with the arm-pull only and with the leg kicking only. Hand/foot force and swimming velocity were measured. The Higuchi’s fractal dimension (HFD) and sample entropy (SampEn) were used for the nonlinear analysis of force and velocity. Both the arm-pull and leg kicking alone were found to produce similar peak and mean hand/foot forces as swimming the full-body stroke. Hand force was more complex in breaststroke and butterfly stroke; conversely, kicking conditions were more complex in front crawl and backstroke. Moreover, the arm-pull and kicking alone tended to be more complex (higher HFD) but more predictable (lower SampEn) than while swimming the full-body stroke. There was no loss of force production from segmental swimming to the full-body counterpart. In conclusion, the number of segments in action influences the nonlinear behavior of the force produced and, when combining the four limbs, the complexity of the hand/foot force tends to decrease.

## Introduction

One of the major limitations of swimming velocity is the swimmer’s ability to produce a thrust. In all four competitive swimming strokes, the segmental actions by the limbs, namely the arm stroke of the upper limbs and leg kicking of the lower limbs, generate hands and feet forces that, summed up with the lift forces generated by hand sculling, result in propelling the body forward, i.e., producing a thrust (Dickinson, 1996; Toussaint, 2000). Each swim stroke presents unique underwater upper- and lower-limbs phases and motor paths (Schleihauf et al., 1988), that added up to the changes in the hands and feet speed (Bilinauskaite et al., 2013b; Gourgoulis et al., 2015), orientation (Bilinauskaite et al., 2013a; Szczepan et al., 2018) and geometry (Bilinauskaite et al., 2013b; Marinho et al., 2009, 2010) throughout the cycle will lead to different force-time profiles (Takagi and Sanders, 2002). However, the characteristics of human swimming produce flows that are very difficult to study due to their nonlinear and turbulent nature (Arellano, 1999; Toussaint and Truijens, 2005), both numerically and experimentally.

Although there are other force components that may come into consideration (such as lift forces), the pressure component is reported as the main contributor to the propulsive force (Samson et al., 2017). Thus, differential pressure systems have proved to be useful assessing more ecologically the effective force produced by each hand/foot individually, including the changes on the force produced over each stroke cycle during actual swimming (Havriluk, 2004, 2009; Santos et al., 2021, 2022b). To the best of our knowledge, there is no study in the literature that comprises the analysis and comparison of the partial contribution of different limbs to full-body propulsion in all swimming strokes.

One can argue that human locomotion in water is a complex, dynamic and nonlinear phenomenon (Barbosa et al., 2016) where a chain of interactions between various components of the stroke affects the hand/foot force separately and concurrently. Thus, minor variations in the force produced within each stroke cycle and between stroke cycles that may go unnoticed by linear analysis, may have a significant impact on swimming performance. As such, nonlinear analyses are gaining traction. Nonlinear analyses look up to nonlinear relationships within the time-data series, i.e., relationships where the output is not linearly correlated with the input, thus linear analyses fail to recognize them (Shi et al., 2020). Furthermore, nonlinear analyses are susceptible to small changes that might have a significant effect on a certain outcome (Lake et al., 2002). Selected nonlinear analyses can identify and quantify the existence of certain properties of a regular time-series, such as persistence (the tendency to repeat a given sequence); or scale invariance (a tendency for a signal to have the same structure when observed on different temporal or spatial scales) (Neumeister et al., 2004). Two of the most common ways to assess these nonlinear properties of a time-data series are by computing its entropy and fractal dimension. Fractal dimension (FD) analyzes the complexity of a pattern (Mandelbrot, 1982). In a time-series, the higher the FD, the higher the complexity of a data-set. Entropy, on the other hand, is a measure of chaos, randomness and uncertainty. In a time-series, high entropy indicates low self-similarity within the data-set, thus less regularity and more randomness (Pincus, 1991). Some studies have chosen nonlinear analysis to assess the influence of speed (Thomas et al., 2017; Wuehr et al., 2013), time spent on the activity (Schiffman et al., 2009; Thomas et al., 2017), constraints (Ahmadi et al., 2019; Schiffman et al., 2009; Sekine et al., 2002; Wuehr et al., 2013), expertise (Preatoni et al., 2010) and disease (Sekine et al., 2002) on the nonlinear behavior of the human gait on land. As far as the sport settings are concerned, researchers are starting to study individual and team tactical expertise resorting to the nonlinear analysis (Gonzalez-Artetxe et al., 2021; Low et al., 2020). In human swimming, as far as our understanding goes, the only variable studied under the light of the nonlinear analysis was velocity. In those studies, authors showed that swimming velocity not only had nonlinear properties but also highly correlated with classical performance-related variables, for example, speed fluctuation (Barbosa et al., 2015a). In other studies, breaststroke and butterfly were found to have more complex velocity patterns compared to front crawl and backstroke (Barbosa et al., 2016, 2017; Bartolomeu et al., 2018). Despite being more complex, the velocity patterns of breaststroke and butterfly were found to be less random and thus, more predictable (lower entropy) (Bartolomeu et al., 2018). Also, the number of limbs in action was reported to influence the entropy and fractal dimension of the velocity (Bartolomeu et al., 2018). Non-expert swimmers’ velocity was reported to have a higher FD (to be more complex) compared to expert and highly qualified athletes (Barbosa et al., 2017), and significantly decreased its complexity after a 100-m all-out bout (Barbosa et al., 2018).

It is yet unclear how different task constraints such as swim conditions (full-body stroke, only arm-pull and only kicking) may influence the hand/foot force-time profiles and thus, the nonlinear analyses’ results. Furthermore, unlike swimming velocity, there is no solid body of knowledge on the feet propulsion and the influence of the lower-limb action on the full-body propulsion. Therefore, it was the aim of this study to examine the nonlinear properties of those forces and describe their variations across the four competitive swimming strokes performing segmental and full-body swimming. It was hypothesized, in line with the findings reported in the literature for swimming velocity (Bartolomeu et al., 2018; Morouço and Barbosa, 2019; Morris et al., 2017), that the force produced in the segmental actions alone would be smaller than the one observed in the full-body stroke. Furthermore, the use of only the upper or lower limbs might influence the limb’s actions, thus making the force production less complex in the segmental strokes than in the full-body stroke. A better understanding of this phenomenon can bring new insights into the locomotion of humans in a challenging and unnatural environment to them. The notion of the complexity of the swimming strokes’ segmental propulsion as well as the characterization of the hand and foot forces across strokes and conditions may help design evidence-based teaching strategies and provide coaches with another tool to assist swimmers in enhancing their performance in water.

## Methods

### Participants

Fifteen male swimmers, with training volumes of approximately 16,000 m per week, took part in this research (age: 16.0 ± 2.9 years, body height: 1.69 ± 0.08 m, body mass: 62.5 ± 14.6 kg). Their personal bests were 61.60 s, 70.15 s, 79.50 s, and 71.7 s at front crawl, backstroke, breaststroke and butterfly, respectively (which correspond to 77 ± 7%, 74 ± 7%, 72 ± 7% and 70 ± 9% of the front crawl, backstroke, breaststroke and butterfly world record, respectively). As inclusion criteria it was set that all participants should be local and/or national level competitors in the two previous seasons, couldn’t have suffered from any musculoskeletal injury in the past 6 months and they agreed beforehand to attend the four scheduled sessions of this study.

All procedures were in accordance with the Helsinki Declaration regarding human research, and the University’s Institutional Review Board approved the research design (Nr: 72/2022). All coaches, parents/guardians and swimmers gave their informed written consent for participation in this study.

### Protocol

Swimmers were randomly assigned into two different groups. Each group performed 16 bouts divided into three consecutive days at the same time of the day. On each day, the swimmer performed from 5 to 6 trials. The second group was asked to be at the pool shore at about the time the first group was foreseen to finish, to avoid long waiting periods at the pool.

Prior to the data collection, all swimmers performed a standard warm-up as described in the literature (Neiva et al., 2014). All swimmers performed four randomly assigned all-out bouts of 25 m in each swim stroke under three different conditions: full-body stroke, arm-pulls only (AO variable) and leg kicking only (KO variable). Given that only a pair of sensors was available, swimmers had to perform the full-body stroke condition twice to allow the collection of the arm-pull values (FA variable) and leg kicking values (FK variable). All bouts started with an in-water push-off and swimmers were instructed to start swimming straight away. Each swimmer had a resting period of 30 min between subsequent bouts. To minimize drag, drafting and other confounding factors, the lanes next to the one where the test was conducted were empty.

### Data Collection

To measure the hands and feet forces while swimming, a differential pressure system was used. The system was composed of two independent sensors (Aquanex Type A, Swimming Technology Research, Inc., Richmond, USA) connected by cabling to an interface that processed the signal (*f* = 100 Hz). Each sensor measured the pressure differential between the front and back planes of the sensors, which were located in palm and dorsum surfaces of the hand/foot, respectively. Despite the system only measured the pressure acting perpendicularly to the planes, being the pressure component the main component of the hand propulsive force (Samson et al., 2017), it was assumed that the former could be representative of the latter.

Prior to each bout, swimmers were asked to calibrate the system: under the arm-stroke conditions, they were asked to keep their hands immersed at the waist level for 10 s (Morais et al., 2023) and under the leg kicking conditions, swimmers were asked to sit at the pool shore with both feet underwater for 10 s. Under both arm-pull conditions, sensors were placed between the proximal phalanges of the 3^rd^ and 4^th^ fingers of both hands. This location is assumed as being a good proxy for the application point of the propulsion vector on the hand (Gourgoulis et al., 2013). Under the leg kicking conditions, sensors were placed between the 2^nd^ and 3^rd^ toes of both feet. Although a few different pressure sensors have been used to assess the hand force (Koga et al., 2022; Lopes et al., 2023; Takagi and Sanders, 2002), those sensors may present some disadvantages: the use of more than one sensor on each hand/foot may change the geometry and volume of the hand, impacting the ecological validity of the propulsion data. Also, additional sensors would mean additional cabling surrounding the limbs which could present a constraint to a free technique. On the other hand, the system used in the present study features a light set-up and was found not to impair nor affect swimming efficiency (Santos et al., 2022b). Furthermore, the present system has been deemed as a reliable method to obtain peak and mean hand resultant force in youth competitive swimmers (Santos et al., 2022a). Simultaneously, swimming velocity was measured. The velocity meter (Swim speedo-meter, Swimsportec, Hildesheim, Germany) features a mechanical system that was placed on the starting block, from which a string comes out and is attached to a belt placed around the swimmer's hip. The system was connected to a 12-bit acquisition card (USB-6008, National Instruments, Austin, USA) which acquired the signal at 50 Hz and transmitted it in real time to a LabView interface (v.2010. National Instruments, Austin, USA). Velocity and force apparatus were video-synchronized. The differential pressure system features a video camera that starts recording at the same time as the sensors start acquiring data and synchronizes both. The velocity meter features a starting light. Force collection started first, the velocity meter started second (with the starting light being captured by the differential pressure system camera) and the swimmers, third. Force data were afterwards synchronized with the starting light of the velocity meter. Both force and speed data were handled afterwards in signal-processing software (AcqKnowledge v.3.7.3, Biopac Systems, Santa Barbara, USA) using a Butterworth 4^th^ order low-pass filter with a 5-Hz cut-off.

### Biomechanical Variables

**Figure 1:**
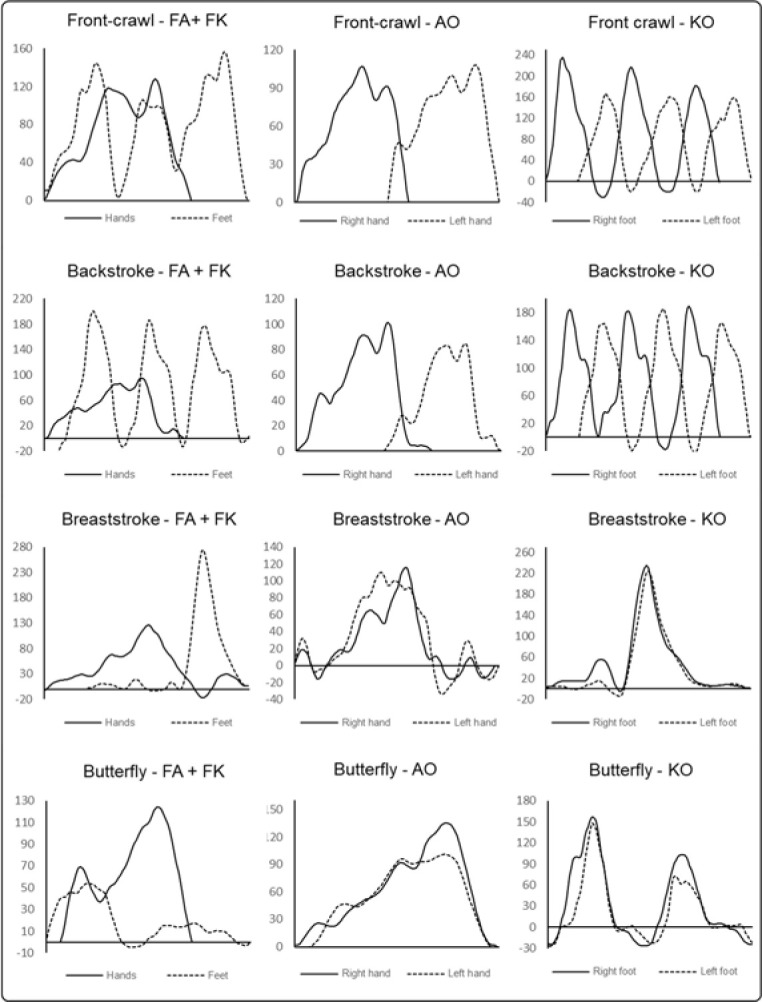
Examples of a time-force curves for each stroke and condition (FA: full stroke with sensors on the hands; FK: full stroke with sensors on the feet; AO: arm-pull only; KO: kicking only), measured in Newtons for a full cycle duration.

For each condition, peak and mean force were calculated (F_pk_ and F_m_, respectively). During kicking in the front crawl, backstroke and butterfly the pressure sensors interpreted the downward and upward kicks as positive and negative force values, thus, the Root Mean Square of the time-series was calculated to avoid biases in F_m_. The arms’ recovery phase was also discarded. Figure 1 depicts an example of typical time-force curves for each stroke and condition. As velocity is related to the force produced, higher force values are expected to emerge with higher speed. To ensure that differences in force production between strokes and conditions were not influenced by velocity, peak and mean velocity (V_pk_ and V_m_, respectively) were also assessed and used as a covariate for the force analysis. In both analyses the first five meters and the last meter were discarded from the analysis, as the push-off and the finish might elicit high variances in the motor behavior that are not caused by the swim stroke itself (Morais et al., 2022).

### Nonlinear Analyses

The Higuchi’s fractal dimension (Higuchi, 1988) was computed to assess the fractal dimension of hand/foot force (F_HFD) and swimming velocity (V_HFD) on a MatLab routine (v.R20013a, MathWorks, Natick, USA) according to the literature (Bartolomeu et al., 2018). As HFD is sensitive to the length of the time-series (*N*),itwas set to be 500 data-time pairs. The tunning variable k-max was determined by plotting the fractal dimension values over a range of various k-max values. The point at which the HFD presented a plateau was considered the saturation point. The mean saturation point was obtained using a k-max value of 60.

Entropy of swimming hand/foot force and swimming velocity were calculated using sample entropy (F_SampEn and V_SampEn, respectively). Sample entropy was computed according to the Richman and Moorman’s (2000) algorithm on a MatLab routine (v.R20013a, MathWorks, Natick, USA) and calculated as described in the literature (Bartolomeu et al., 2018). Data length was set to be higher than 500 speed-time pairs, which according to the literature reduces the bias of correlation templates (Richman and Moorman, 2000; Yentes et al., 2013). The length of sequences to be compared and the tolerance value were set to be m = 2 and r = 0.2, respectively. These values were denoted as an appropriate choice for the type and length of data under analysis (Chen et al., 2009; Richman and Moorman, 2000).

### Statistical Analyses

The normality of the data distribution was tested using the Shapiro-Wilk test. Mean ± 1 SD are reported for all dependent variables alongside with the 95% confidence intervals (95CI). Variations across swimming strokes and conditions were analyzed resorting a univariate analysis of covariance (ANCOVA). This analysis was performed for both Fpk and Fm separately whilst statistically controlling for velocity. When the interaction effect was statistically significant, a follow-up analysis with Bonferroni correction was carried out to assess the simple effects. The significance level was set at *p*< 0.05. Effect size of the variance was reported resorting to the Partial Eta-Squared (η_p^2) and was considered: (1) minimum if 0.02 < η_p^2 ≤ 0.13; (2) moderate if 0.13 ≤ η_p^2 < 0.26; and (3) strong if η_p^2 ≥ 0.26 (Cohen, 1988). Pearson´s correlation (r) was also carried out to assess the association between kinetic, kinematic and nonlinear variables (*p* ≤ 0.05). Correlation effect sizes were interpreted as null if 0 < |r| ≤ 0.1, small if 0.1 < |r| ≤ 0.3, moderate if 0.3 < |r| ≤ 0.5 and strong if |r| > 0.5. All statistical procedures were performed in a statistical software package (SPSS v.21, IBM, New York, USA).

## Results

### Hand/Foot Force

There were significant and moderate stroke x condition interactions in both F_pk_ and F_m_(F_9,139_ = 2.145, *p* = 0.013, $\eta_{p}^{2}$ = 0.135 and F_9,139_ = 2.844, *p* = 0.004, $\eta_{p}^{2}$ = 0.156, respectively) (Table 1). The post-hoc test showed that F_pk_ was significantly different between strokes (*p*< 0.001), except for front crawl vs. backstroke, front crawl vs. butterfly and backstroke vs. butterfly. Overall, whilst controlling for velocity (Madj ± SE rows in Table 1), F_pk_ was higher in breaststroke, followed-up by backstroke, front crawl and butterfly. FA and AO conditions presented no significant differences in F_pk_. On the other hand, FK was significantly lower than KO. In fact, KO condition was significantly higher than the other variants. All these results were obtained whilst controlling for velocity. Regarding F_m_ (still controlling for velocity), the post-hoc test showed a significant difference between butterfly and breaststroke. Breaststroke presented larger values, followed-up by front crawl, backstroke and butterfly. The full-body conditions (FA and FK) were significantly smaller than segmental conditions (AO and KO, respectively). Fpk values were controlled for a velocity of 1.54m•s^−1^ and Fm values for a velocity of 1.03m•s^−1^.

**Table 1:** Interactions and main effects of stroke and condition on peak force (F_pk_) and mean force (F_m_) whilst controlling for velocity.

	Peak Force (F_pk_)				Mean Force (F_m_)			
	df	F-ratio	*p*	$\eta_{p}^{2}$	df	F-ratio	*p*	$\eta_{p}^{2}$
Stroke	3, 139	16.867	<0.001	0.267	3, 135	4.439	0.005	0.087
Condition	3, 139	9.854	<0.001	0.175	3, 135	3.921	0.010	0.078
Stroke x Condition	9, 139	2.145	0.013	0.135	9, 135	2.844	0.004	0.156

In absolute values, i.e., not controlling for velocity (M ± SD rows in Table 1), both AO and KO presented F_pk_ and F_m_ values around 100% of the FA and FK, respectively (Table 2). The exceptions were backstroke F_pk_ at KO (84%), and butterfly F_pk_ and F_m_ at KO (135% and 131%, respectively). Whereas, controlling for velocity, both segmental conditions had F_pk_ and F_m_ values above 100% (with the exception for 96% for Fm at the Front-crawl’s AO condition) of the full-body stroke conditions.

**Table 2:** Means (M), Adjusted Means (Madj), Standard Deviations (SD) and Standard Errors (SE) for hand/foot force across the four strokes for the full-body conditions. Percentage of the force of segmental conditions in relation to the full stroke.

			FA	% FA	FK	% FK
Front crawl	F_pk_ (N)	M ± 1 SD	97.85 ± 40.42	100	110.31 ± 68.75	100
		Madj ± SE	81.56 ± 13.62	100	94.09 ± 13.36	100
	F_m_ (N)	M ± 1 SD	34.40 ± 16.22	100	36.45 ± 29.23	100
		Madj ± SE	29.07 ± 4.49	100	31.23 ± 4.49	100
Backstroke	F_pk_ (N)	M ± 1 SD	76.42 ± 16.72	100	115.15 ± 34.045	100
		Madj ± SE	80.34 ± 13.44	100	123.19 ± 13.69	100
	F_m_ (N)	M ± 1 SD	25.53 ± 6.51	100	37.61 ± 19.82	100
		Madj ± SE	19.21 ± 4.77	100	33.17 ± 4.70	100
Breaststroke	F_pk_ (N)	M ± 1 SD	120.02 ± 37.66	100	194.62 ± 51.33	100
		Madj ± SE	98.61 ± 12.99	100	178.27 ± 12.80	100
	F_m_ (N)	M ± 1 SD	31.70 ± 11.19	100	29.08 ± 8.87	100
		Madj ± SE	31.02 ± 4.01	100	29.37 ± 4.01	100
Butterfly	F_pk_ (N)	M ± 1 SD	110.01 ± 43.63	100	77.60 ± 31.96	100
		Madj ± SE	84.59 ± 15.44	100	59.15 ± 14.96	100
	F_m_ (N)	M ± 1 SD	34.76 ± 15.20	100	16.03 ± 4.97	100
		Madj ± SE	28.69 ± 5.03	100	13.20 ± 5.06	100

FA – Full-stroke arm-pull; FK – Full-stroke kicking; AO – Arm-pull only; KO – Kicking only; F_pk_ adjusted for the mean peak velocity v = 1.54 m•s^−1^; F_m_ adjusted for the mean velocity v = 1.03 m•s^−1^

### Higuchi’s Fractal Dimension (HFD)

A significant and strong stroke x condition interaction was noted (F_9,117_ = 10.546, *p* < 0.001, $\eta_{p}^{2}$ = 0.448) (Table 3). A significant and strong main effect of swimming stroke was also found (F_3,39_ = 10.080, *p*< 0.001, $\eta_{p}^{2}$ = 0.456) (Table 3). The post-hoc test showed that backstroke’s force production was significantly less complex (lower F_HFD) than the force produced while swimming the other strokes (0.001 <*p*< 0.046). Next strokes were front crawl, breaststroke and butterfly, respectively. Another significant and strong main effect was found for swimming condition (F_3,39_ = 73.889, *p*< 0.001, $\eta_{p}^{2}$ = 0.850). All conditions, apart from the pairwise FA vs. AO, were significantly different in the post-hoc test (*p*< 0.001). All swimming strokes exhibited the same behavior in the conditions under study, i.e., the most complex force production (highest F_HFD) was showed by the KO condition, followed-up by FK, AO and FA.

**Table 3:** Means (M), Adjusted Means (Madj), Standard Deviations (SD) and Standard Errors (SE) for hand/foot force across the four strokes for the segmental conditions. Percentage of the force of segmental conditions in relation to the full stroke.

			AO	% FA	KO	% FK
Front crawl	F_pk_ (N)	M ± 1 SD	95.49 ± 36.13	98	103.45 ± 49.50	94
		Madj ± SE	99.92 ± 12.92	124	131.67 ± 13.71	140
	F_m_ (N)	M ± 1 SD	31.76 ± 15.63	92	35.21 ± 27.11	97
		Madj ± SE	27.99 ± 4.44	96	42.31 ± 4.57	135
Backstroke	F_pk_ (N)	M ± 1 SD	75.82 ± 20.13	99	96.73 ± 32.42	84
		Madj ± SE	100.84 ± 14.64	124	156.65 ± 15.82	127
	F_m_ (N)	M ± 1 SD	24.31 ± 8.44	95	36.00 ± 11.35	96
		Madj ± SE	24.85 ± 4.63	129	44.23 ± 4.86	133
Breaststroke	F_pk_ (N)	M ± 1 SD	109.32 ± 41.78	91	192.66 ± 53.47	99
		Madj ± SE	125.03 ± 12.92	127	188.82 ± 12.34	106
	F_m_ (N)	M ± 1 SD	31.69 ± 11.78	100	30.51 ± 8.15	105
		Madj ± SE	39.31 ± 4.21	127	37.33 ± 4.21	127
Butterfly	F_pk_ (N)	M ± 1 SD	98.87 ± 45.40	90	104.55 ± 47.73	135
		Madj ± SE	97.29 ± 14.40	115	106.73 ± 14.41	180
	F_m_ (N)	M ± 1 SD	31.91 ± 13.75	92	20.95 ± 13.59	131
		Madj ± SE	30.61 ± 4.91	107	26.02 ± 4.99	197

FA – Full-stroke arm-pull; FK – Full-stroke kicking; AO – Arm-pull only; KO – Kicking only; F_pk_ adjusted for the mean peak velocity v = 1.54 m•s^−1^; F_m_ adjusted for the mean velocity v = 1.03 m•s^−1^

### Sample Entropy

A significant and strong stroke x condition interaction was observed in the F_SampEn (F_9,117_ = 13.446, *p*< 0.001, $\eta_{p}^{2}$ = 0.508) (Table 4). Moreover, a significant and strong effect of swimming stroke was found (F_3,39_ = 61.2, *p*< 0.001, $\eta_{p}^{2}$ = 0.825) (Table 4). Post-hoc tests showed that all strokes were significantly different from each other (0.001 <*p*< 0.003) except front crawl vs. butterfly. Breaststroke was the stroke with less randomness (lower SampEn), followed-up by backstroke, front crawl and butterfly.

The FK condition presented the highest values in all swimming strokes, with the exception of breaststroke, where FK and KO noted the lowest values in the whole test. FK was significantly higher than the KO condition.

**Table 4:** Mean Higuchi’s fractal dimension (HFD) of hand/foot force across the four strokes and conditions, standard deviations (SD) and confidence intervals (CI); Interactions and main effects of the HFD.

Descriptive				
	FA M ± 1 SD (95CI)	FK M ± 1 SD (95CI)	AO M ± 1 SD (95CI)	KO M ± 1 SD (95CI)
Front crawl	1.928± 0.012^a^ (1.921;1.935)	1.955± 0.013^b^ (1.947;1.963)	1.938± 0.009^a^ (1.932;1.943	1.967± 0.008^c^ (1.963;1.972)
Backstroke	1.917 ± 0.013^a^ (1.910;1.925)	1.956 ± 0.013^b^ (1.948;1.963)	1.922 ± 0.013^a^ (1.914;1.929	1.969± 0.005^c^ (1.966;1.971)
Breaststroke	1.940 ± 0.012^a^ (1.933;1.947)	1.954 ± 0.010^bc^ (1.947;1.960)	1.946 ± 0.009^ab^ (1.94;1.952	1.961 ± 0.006^c^ (1.958;1.965)
Butterfly	1.941 ± 0.011^a^ (1.934;1.947)	1.957 ± 0.010^bc^ (1.951;1.963)	1.947 ± 0.011^ab^ (1.941;1.954)	1.959 ± 0.013^c^ (1.951;1.967)
ANCOVA				
	df	F-ratio	*p*	$\eta_{p}^{2}$
Stroke	3, 39	10.080	<0.001	0.456
Condition	3, 39	73.889	<0.001	0.850
Stroke x condition	9, 117	10.546	<0.001	0.448

FA – Full-stroke arm-pull; FK – Full-stroke kicking; AO – Arm-pull only; KO – Kicking only; For each swimming stroke (i.e., for each line), if two conditions have the same superscript letter (a, b or c) they are not significantly different and if two conditions have different superscript letters (a, b or c) they are significantly different; (*p*<0.05).

### Correlation Matrixes

Peak and mean hand/foot forces (F_pk_ and F_m_) were strongly correlated (r > 0.665), as well as, mean and peak velocities (V_pk_ and V_m_, moderate/strong correlation: 0.385 < r < 0.586) (Table 5). The correlation between velocity and force was more noticeable for the pairs F_pk_ x V_pk_ and F_m_x V_m_ (0.252 < r < 0.402 and 0.261 < r < 0.308, respectively) (Table 5).

**Table 5:** Mean Sample entropy (SampEn) of hand/foot force across the four strokes and conditions, standard deviations (SD) and confidence intervals (CI); Interactions and main effects of the SampEn.

Descriptive					
	FA M ± 1 SD (95CI)	FK M ± 1 SD (95CI)	AO M ± 1 SD (95CI)		KO M ± 1 SD (95CI)
Front crawl	0.333 ± 0.070^a^ (0.292;0.374)	0.344 ± 0.103^a^ (0.284;0.403)	0.322 ± 0.077^a^ (0.278;0.367)		0.295 ± 0.115^a^ (0.228;0.361)
Backstroke	0.245 ± 0.075^a^ (0.201;0.288)	0.348 ± 0.063^b^ (0.310;0.384)	0.244 ± 0.055^a^ (0.212;0.276)		0.303 ± 0.069^ab^ (0.263;0.343)
Breaststroke	0.208 ± 0.088^a^ (0.157;0.258)	0.114 ± 0.039^b^ (0.091;0.137)	0.243 ± 0.064^a^ (0.206;0.280)		0.104 ± 0.037^b^ (0.083;0.125)
Butterfly	0.322 ± 0.056^a^ (0.289;0.354)	0.399 ± 0.069^b^ (0.359;0.439)	0.321 ± 0.053^a^ (0.291;0.352)		0.352 ± 0.079^ab^ (0.307;0.397)
ANCOVA					
	df	F-ratio	*p*		$\eta_{p}^{2}$
Stroke	3, 39	61.200	<0.001		0.825
Condition	3, 39	1.759	0.171		0.119
Stroke x condition	9, 117	13.446	<0.001	0.508	

FA – Full-stroke arm-pull; FK – Full-stroke kicking; AO – Arm-pull only; KO – Kicking only; For each swimming stroke (i.e., for each line), if two conditions have the same superscript letter (a or b) they are not significantly different and if two conditions have different superscript letters (a or b) they are significantly different; (*p*< 0.05).

Peak force (F_pk_) was moderately and negatively correlated to both velocity’s (0.378 < r < 0.411) and force’s (0.301 < r < 0.467) sample entropy. Peak velocity (V_pk_) was moderately and negatively correlated to V_SampEn whilst controlling for stroke x condition; and V_m_ to F_HFD when not controlling and controlling for stroke (Table 6).

**Table 6:** Pearson’s product-moment correlation between kinematic and kinetic variables.

		F_pk_ (N)			F_m_ (N)			V_pk_ (m•s^−1^)		
		*df*	*r*	*p*	*df*	*r*	*p*	*df*	*r*	*p*
No Control	F_m_ (N)	224	0.728	<0.001^†^	-----	-----	-----	-----	-----	-----
	V_pk_ (m•s^−1^)	156	0.273	0.001*	156	0.139	0.160	-----	-----	-----
	V_m_(m•s^−1^)	156	0.048	0.555	156	0.268	0.001*	156	0.551	<0.001^†^
Controlling for stroke	F_m_ (N)	153	0.697	<0.001^†^	-----	-----	-----	-----	-----	-----
	V_pk_ (m•s^−1^)	153	0.252	0.002*	153	0.251	0.002*	-----	-----	-----
	V_m_(m•s^−1^)	153	0.056	0.486	153	0.261	0.001*	153	0.586	<0.001^†^
Controlling for condition	F_m_ (N)	153	0.665	<0.001^†^	-----	-----	-----	-----	-----	-----
	V_pk_ (m•s^−1^)	153	0.402	<0.001^‡^	153	0.216	0.007*	-----	-----	-----
	V_m_(m•s^−1^)	153	0.147	0.068	153	0.308	<0.001^‡^	153	0.385	<0.001^‡^
Controlling for stroke x condition	F_m_ (N)	152	0.706	<0.001^†^	-----	-----	-----	-----	-----	-----
	V_pk_ (m•s^−1^)	152	0.385	<0.001^‡^	152	0.285	<0.001*	-----	-----	-----
	V_m_(m•s^−1^)	152	0.158	0.050*	152	0.300	<0.001^‡^	152	0.426	<0.001^‡^

F_pk_ – peak force; F_m_ – mean force; V_pk_ – peak velocity; V_m_ – mean velocity; Significant with: * – small effect size; ^‡^ – moderate effect size; ^†^ – strong effect size.

**Table 7:** Pearson’s product-moment correlation between kinematics, kinetics and selected nonlinear variables.

		F_pk_ (N)			F_m_ (N)			V_pk_ (m•s^−1^)			V_m_(m•s^−1^)		
		*df*	*r*	*p*	*df*	*r*	*p*	*df*	*r*	*p*	*df*	*r*	*p*
No Controlling	F_SampEn (a.u.)	224	−0.301	<0.001^‡^	224	0.149	0.026*	156	0.015	0.850	156	0.218	0.006*
	F_HFD (a.u.)	224	0.137	0.041*	224	−0.036	0.596	156	−0.204	0.011*	156	−0.371	<0.001^‡^
	V_SampEn (a.u.)	156	−0.411	<0.001^‡^	156	0.067	0.408	156	−0.203	0.011*	156	0.291	<0.001*
	V_HFD (a.u.)	156	0.076	0.346	156	−0.197	0.013*	156	0.257	0.001*	156	−0.115	0.151
Controlling for stroke	F_SampEn (a.u.)	153	−0.467	<0.001^‡^	153	0.018	0.826	153	0.040	0.619	153	0.214	0.008*
	F_HFD (a.u.)	153	0.119	0.139	153	−0.079	0.331	153	−0.229	0.004*	153	−0.368	<0.001^‡^
	V_SampEn (a.u.)	153	−0.395	<0.001^‡^	153	0.018	0.828	153	−0.147	0.068	153	0.284	<0.001*
	V_HFD (a.u.)	153	0.028	0.731	153	−0.136	0.092	153	0.177	0.027*	153	−0.098	0.227
Controlling for condition	F_SampEn (a.u.)	153	−0.467	<0.001^‡^	153	0.034	0.674	153	−0.036	0.661	153	0.202	0.012*
	F_HFD (a.u.)	153	0.061	0.448	153	−0.099	0.222	153	0.096	0.235	153	−0.115	0.155
	V_SampEn (a.u.)	153	−0.395	<0.001^‡^	153	0.065	0.421	153	−0.360	<0.001^‡^	153	0.222	0.006*
	V_HFD (a.u.)	153	0.093	0.250	153	−0.200	0.012*	153	0.234	0.003*	153	−0.207	0.010*
Controlling for stroke x condition	F_SampEn (a.u.)	152	−0.461	<0.001^‡^	152	0.017	0.838	152	−0.009	0.916	152	0.196	0.015*
	F_HFD (a.u.)	152	0.051	0.530	152	−0.084	0.299	152	0.074	0.359	152	−0.109	0.179
	V_SampEn (a.u.)	152	−0.378	<0.001^‡^	152	0.015	0.854	152	−0.305	<0.001^‡^	152	0.209	0.009*
	V_HFD (a.u.)	152	0.046	0.573	152	−0.139	0.086	152	0.135	0.094	152	−0.193	0.016*

**Table 7(cont.):** Pearson’s product-moment correlation between kinematics, kinetics and selected nonlinear variables.

		F_SampEn (a.u.)			F_HFD (a.u.)			V_SampEn (a.u.)		
		*df*	*r*	*p*	*df*	*r*	*p*	*df*	*r*	p
No Controlling	F_SampEn (a.u.)	-----	-----	-----	-----	-----	-----	-----	-----	-----
	F_HFD (a.u.)	224	0.101	0.131	-----	-----	-----	-----	-----	-----
	V_SampEn (a.u.)	156	0.533	<0.001^†^	156	−0.182	0.023*	-----	-----	-----
	V_HFD (a.u.)	156	−0.259	<0.001*	156	−0.045	0.574	156	−0.259	<0.001*
Controlling for stroke	F_SampEn (a.u.)	-----	-----	-----	-----	-----	-----	-----	-----	-----
	F_HFD (a.u.)	221	0.108	0.108	-----	-----	-----	-----	-----	-----
	V_SampEn (a.u.)	153	0.529	<0.001^†^	153	−0.169	0.036*	-----	-----	-----
	V_HFD (a.u.)	153	−0.242	0.002*	153	−0.082	0.308	153	−0.169	0.036*
Controlling for condition	F_SampEn (a.u.)	-----	-----	-----	-----	-----	-----	-----	-----	-----
	F_HFD (a.u.)	221	0.152	0.023*	-----	-----	-----	-----	-----	-----
	V_SampEn (a.u.)	153	0.528	<0.001^†^	153	−0.089	0.269	-----	-----	-----
	V_HFD (a.u.)	153	−0.272	<0.001*	153	0.017	0.832	153	−0.289	<0.001*
Controlling for stroke x condition	F_SampEn (a.u.)	-----	-----	-----	-----	-----	-----	-----	-----	-----
	F_HFD (a.u.)	220	0.161	0.017*	-----	-----	-----	-----	-----	-----
	V_SampEn (a.u.)	152	0.524	<0.001^†^	152	−0.068	0.400	-----	-----	-----
	V_HFD (a.u.)	152	−0.256	<0.001*	152	−0.020	0.802	152	−0.202	0.012*

F_pk_ – peak force; F_m_ – mean force; V_pk_ – peak velocity; V_m_ – mean velocity; F_SampEn – Sample entropy of hand/foot force; F_HFD – Fractal dimension of hand/foot force; V_SampEn − Sample entropy of velocity; V_HFD - Fractal dimension of velocity; a.u. – arbitrary units; Significant with: * – small effect size; ^‡^ – moderate effect size and; † – large effect size.

Regarding the nonlinear behavior of velocity and hand/foot force, V_SampEn was strongly correlated to F_SampEn for every controlling condition (0.524 < r < 0.533) (Table 6).

## Discussion and Implications

The aim of this study was to examine the force produced by the swimmers’ hands and feet while free swimming, and its nonlinear behavior performing the full-body stroke, only the arm-pull, and only the leg kicking. It was shown that, in absolute values, the force produced by the limbs under the partial conditions fell around 100% or more (exception made for backstroke F_pk_ at KO) than the value observed for the full-body stroke. Both hand and foot forces presented nonlinear properties that were significant and moderately affected by the swimming stroke and condition under analysis.

### Segmental Action and Force Production

While swimming, the force generated varies over stroke cycles (Hollander et al., 1986). Thus, one should consider instantaneous force values as well as mean values in order to ensure a comprehensive study of swimming performance. In the present study, peak and mean values were assessed. Peak and mean force are deemed as convenient and straightforward variables when characterizing in-water human locomotion (Barbosa et al., 2020).

Values reported in the literature using the same differential pressure system fall around those in the present study for front crawl (Morais et al., 2020, 2022), breaststroke (Werlang et al., 2017) and butterfly (Pereira et al., 2015). Differences in peak and mean values can be attributed to the different competitive level of swimmers and their stroke specialization. Elite swimmers are faster than their non-elite counterparts. As force is related to velocity, one could expect, at least, higher Fm values. Furthermore, if a swimmer is specialized in a specific swimming stroke, he/she may be more efficient in force production, thus making Fpk and/or Fm values vary. At slower velocities, unsurprisingly, smaller absolute force values were reported (Barbosa et al., 2020; Ng et al., 2020). There is no record for backstroke using the same apparatus. With a different set of sensors, the results reported were somewhat lower than those found in the present study (Kudo et al., 2013; Takagi and Sanders, 2002). However, authors have analyzed swimmers at 80% of their personal best. Nonetheless, and interestingly, in their studies higher peak forces were observed at breaststroke, followed by butterfly, front crawl and backstroke, which is in accordance with the present study. Values reported in the literature for tethered swimming, on the other hand, are generally higher (Gomes et al., 2018; Morouço et al., 2015a; Yeater et al., 1981). It is important to note that differences between applied methodologies may explain the different results: while the tethered method measures the sum of forces acting on the body (i.e., the force with which the swimmer pulls a string connected to a load cell), the differential pressure system measures the pressure component produced by the hands/feet acting perpendicularly to the sensors (Santos et al., 2021).

There is evidence that, despite arm-pull and kicking alone have slower velocities than the full-body stroke, the sum of both velocity values is far greater than the one observed in full-body stroke (Bartolomeu et al., 2018; Morouço and Barbosa, 2019; Morris et al., 2017). Likewise, for tethered swimming it is also reported in the literature that the sum of the segmental strokes’ thrust is greater than the thrust of the full-body stroke (Morouço et al., 2015b; Morouço and Barbosa, 2019; Yeater et al., 1981). Overall, the literature suggests that arms-legs synchronization is a challenging task constraint for humans that leads to a loss of effectiveness. Yeater et al. (1981) suggested that turbulence or flow from the arm-pull would reduce the force produced by kicking. However, Gourgoulis et al. (2014) reported that at front crawl, the addition of a flutter kick to the full-body stroke did not influence the effectiveness of the arm-pull and the present study found that, for absolute values (i.e., not controlling for velocity), both kicking alone and the arm-pull alone produced, in general, similar or higher Fpk and Fm compared to when swimming the full-body stroke. In fact, only backstroke Fpk at KO presented a decrease in force production greater than 10% (16%). Thereby, one can wonder that the loss of velocity from the sum of the segmental actions to the full-body stroke, studied for decades, is not due to significant loss in force production. Thus, the arm-pull flow canceling out the kicking force production does not seem to be the best explanation available. It is drag force that seems to be one of the causes. Based on a computational simulation, it was reported that when adding kicking to the arm-pull, there is a tangential drag force in kicking that counteracts the thrust produced by the lower limbs (Nakashima, 2007). The present findings agree with this hypothesis and add experimental insights into the argument. Thus, kicking produces a meaningful amount of force, however, a good portion of it is not translated forwards to the overall thrust.

Referring to Table 2, one can notice that variations of Fpk and Fm across strokes were similar two-by-two: the behavior was similar for the pairs FA/AO and FK/KO across strokes. In tandem, Gourgoulis et al. (2014) reported no changes between FA and AO conditions for the magnitude of the propulsive drag and thrust. They put forward that neither the pitch and sweepback angles, nor the magnitude of the hand’s velocity were significantly changed when kicking was performed. Combining the findings by Gourgoulis et al. (2014) and the results of the present study, one might presume that the addition of the legs to the full-body stroke does not alter the hands’ kinetics, or at least, possible changes are not reflected in the hands’ angles, velocity and force production.

### Nonlinear Properties of Force Production

The FD of the human gait on land has been reported to decrease with the time spent on the activity (Schiffman et al., 2009), increase from level walking to upstairs walking (Sekine et al., 2002), increase from preferred walking velocity to maximal walking velocity (Wuehr et al., 2013) and increase from walking with eyes open to walking with eyes closed (Schiffman et al., 2009; Sekine et al., 2002; Wuehr et al., 2013). In swimming, HFD of velocity was reported to decrease under fatigue (Barbosa et al., 2018) and is prone to be lower in expert swimmers (Barbosa et al., 2017). Also, HFD of velocity was found to be smaller at front crawl and backstroke than breaststroke and butterfly stroke (Barbosa et al., 2016, 2017; Bartolomeu et al., 2018). Barbosa et al. (2016, 2017) and Bartolomeu et al. (2018) argued that a set of constraints could explain these differences. The combination of the amount of resistance in each swim stroke (environmental constraint), the selected combination of stroke rate-stroke length for each swim stroke (task constraint) and specific anthropometric features that are more suitable for a given swim stroke than another (organismic constraint), influence the complexity of swimming velocity. The present study found a similar trend in the hand force’s HFD under both arm-pull conditions. Propulsive force is a strong predictor of swimming velocity, and being the upper limbs the main contributors to velocity (Deschodt et al., 1999; Hollander, 1988), this link between the nonlinear properties of the swim velocity and hand force was somewhat expected.

The most complex force production was noted under the KO condition, followed by FK, AO and FA. These findings denote consistency in the influence (constraint) of the segments in use on the swimming complexity. Being the HFD smaller at front crawl and backstroke than breaststroke and butterfly stroke, under both arm-pull conditions, it seems that the simultaneous nature of the stroke’s pull makes the hand force more complex. Conversely, flutter kicking presented the most complex force pattern, possibly due to the natural body rotation in the transverse axis that swimmers are subjected to. When swimming front crawl and backstroke, kicking is not restricted to a single plane, but occurs in the sagittal and oblique planes (unlike the butterfly, restricted to the sagittal plane, and breaststroke, restricted to the oblique plane) (Maglischo, 2003). This continuous transition between planes can be an explanation for the higher complexity of force production of front crawl and backstroke kicking. This explanation also fits the decrement in complexity from the arm-pull alone to the arm-pull under the full-body stroke condition. Adding the leg kicking has been reported do diminish the body roll (Gourgoulis et al., 2014) when comparing to the arm-pull alone condition, and a lighter body roll may justify smaller complexity under the full-body stroke condition. It must be stressed that the body roll was not measured in the present study, thus results should be interpreted carefully. Interestingly, all swimming strokes exhibited the same pattern of complexity across conditions.

On-land human walking SampEn was reported to increase with fatigue (Thomas et al., 2017), increase with raising visuomotor stimulus (Ahmadi et al., 2019) and to be lower in experts and highly-skilled performers (Preatoni et al., 2010). The results of the present study suggest for the first time the presence of entropy in hand/foot force of humans’ swimming. Literature on the randomness of swimming velocity reports mixed findings (Barbosa et al., 2016, 2017; Bartolomeu et al., 2018). However, different algorithms were used to calculate entropy. It has been claimed that some algorithms are largely dependent on the length of the data-series and may lack relative consistency (Chen et al., 2009; Richman and Moorman, 2000; Yentes et al., 2013). SampEn, on the other hand, does not include self-matches in calculating the conditional probability as approximate entropy does, thus improving relative consistency (Richman and Moorman, 2000). For swimming velocity, backstroke and front crawl were found to have the more random velocity pattern (Bartolomeu et al., 2018). In the present study, it was hypothesized that front crawl and backstroke would be those with more random force production (higher SampEn). The hypothesis was partially confirmed. Under both arm-pull conditions, front crawl was indeed the swim stroke with the largest unpredictability force production, but concurrently to butterfly stroke. Both swim strokes feature similar upper-limb trajectories, which may explain the non-significant differences between the two. Conversely, upper-limbs trajectories in front crawl and backstroke are very different, making the former significantly more random than the latter.

### Influence of Nonlinear Properties on Force Production

In general, the force produced at segmental actions alone was close (when no control was applied) or higher (when controlling for velocity) than at the full-body stroke. Thereby, based on the present findings, one can presume that the difference in velocity, from the sum of the segmental values to the full-body values reported in the literature (Bartolomeu et al., 2018) is not due to a loss in force production. Several explanations have been put forward to explain the loss from the sum of the segmental actions to the full-body stroke (discussed in “the segmental action and force production” subsection). For the first time, nonlinear analysis can help explain or further characterize this phenomenon. First, as seen for velocity (Bartolomeu et al., 2018), in hand/foot force, strokes tend to present higher SampEn under the full-body conditions (except for Breaststroke FA). I.e., the arm-pull was more predictable when swimming with arms only than when swimming the full-body stroke. The same for the leg kicking. This fact highlights the positive and strong correlation found between V_SampEn and F_SampEn under every controlling condition (Table 6), which means that lower hand/foot force predictability will likewise lower the velocity’s predictability.

The negative correlation between V_pk_ x V_SampEn and F_pk_ x F_SampEn found whilst controlling for stroke x condition means that increases in peak velocity and hand/foot force, determinants of an increased performance, can characterize a decrease in SampEn. These results are in agreement with the literature that reports a decrease in entropy over a competitive season while overall performance increased and a decrease in SampEn from experts to highly qualified swimmers (Barbosa et al., 2015b, 2017).

Notwithstanding the increased SampEn at full-body stroke when comparing to segmental velocities, swimmers are still able to reach high velocities, i.e., absolute velocity and absolute force values are still higher in full-stroke than any of the segmental conditions individually. Thus, despite the increase in unpredictability (SampEn), swimmers were able to offset it. This might be due to the reduced complexity. The limbs’ action at full-body stroke (FA and FK conditions) presented a lower intra-cyclic variation (lower HFD value, thus less complex) than their counterparts (AO and KO conditions, respectively). In other words, when joining the four limbs together, the limbs’ actions became more unpredictable but less complex. Interestingly, when controlling for stroke x condition, F_HFD was not related neither with velocity nor with hand/foot force indexes. Thus, one can presume that the act of combining the four limbs was the main factor responsible for decreased complexity. Previous research has attributed leg kicking other roles besides propulsion, such as refinement of whole-body alignment (Cohen et al., 2014; Gonjo et al., 2021; Gourgoulis et al., 2014; Yanai, 2001) and therefore reducing active drag (Seifert et al., 2007). The present results add-on the decrease in the complexity of the force production to the body of knowledge on the function of leg kicking, in all four strokes.

From a teaching perspective, programs often advise pupils to start by learning front crawl and backstroke (Costa et al., 2017). The findings of the present study seem to backup this assumption: the hand and foot force patterns of the swim strokes featuring alternate action by both upper- and lower-limbs (i.e., front crawl and backstroke) were more random than the other two, however, less complex. Thus, if the coach is time-limited, the recurrent teaching model seems appropriate. Nevertheless, as both velocity (Bartolomeu et al., 2018) and force seem to be less complex in backstroke than in front crawl, one could hypothesize to what extent it would be more beneficial to start teaching backstroke in the first place, benefitting from the absence of the breathing/arm-pull coordination constraint. Concerning training, the lack of loss of propulsive force from partial limbs´ actions to the full-body stroke suggests that training of hands/feet propulsive force alone has direct applicability to the full-body stroke. Moreover, in a specific propulsive force session, special attention should be given to AO and KO variants, which are often undervalued, as not only they have a tendency to a more regular pattern (lower entropy) that could be transferred to full-body stroke swimming, but also a more complex propulsive force production (higher FD) that might need more training. Future research should, however, be carried out to address this last presumption, as despite expertise is a factor for lower velocity’s FD (Barbosa et al., 2017), it still remains unclear whether specific segmental training would reduce the complexity of the force pattern for each subject in particular.

## Conclusions

Generally speaking, both the arm-pull and leg kicking alone were found to produce peak and mean hand/foot force, in absolute values, as those observed while swimming with the full-body stroke. The produced force exhibited nonlinear behaviors. Hand force under the arm-pull conditions is more complex in breaststroke and butterfly stroke; conversely, under kicking conditions foot force is more complex in front crawl and backstroke. Moreover, arm-pull and kicking alone tend to be more complex (higher HFD), but more predictable (lower SampEn) than swimming the full-body stroke. When combining the four limbs together, the complexity of the hand/foot force tends to decrease.
